# Identification of a Conserved Prophenoloxidase Activation Pathway in Cotton Bollworm *Helicoverpa armigera*

**DOI:** 10.3389/fimmu.2020.00785

**Published:** 2020-05-05

**Authors:** Qianran Wang, Mengyi Yin, Chuanfei Yuan, Xijia Liu, Zhihong Hu, Zhen Zou, Manli Wang

**Affiliations:** ^1^State Key Laboratory of Virology, Center for Biosafety Mega-Science, Wuhan Institute of Virology, Chinese Academy of Sciences, Wuhan, China; ^2^Savaid Medical School, University of Chinese Academy of Sciences, Beijing, China; ^3^State Key Laboratory of Integrated Management of Pest Insects and Rodents, Institute of Zoology, Chinese Academy of Sciences, Beijing, China; ^4^Key Laboratory of Tropical Translational Medicine, Laboratory of Medicine, School of Tropical Medicine, Ministry of Education, Hainan Medical University, Haikou, China

**Keywords:** melanization, prophenoloxidase, serine protease, baculovirus, *Helicoverpa armigera*

## Abstract

Melanization is a prominent insect humoral response for encapsulation of and killing invading pathogens. It is mediated by a protease cascade composed of a modular serine protease (SP), and clip domain SPs (cSPs), which converts prophenoloxidase (PPO) into active phenoloxidase (PO). To date, melanization pathway in cotton bollworm *Helicoverpa armigera*, an important agricultural pest, remains largely unclear. To biochemically reconstitute the pathway *in vitro*, the putative proteases along with modified proteases containing the factor Xa cleavage site were expressed by *Drosophila* S2 cell expression system. Purified recombinant proteins were used to examine their role in activating PPO. It is revealed that cascade is initiated by a modular SP-SP41, followed by cSP1 and cSP6. The three-step SP41/cSP1/cSP6 cascade could further activate PPO, and the PO activity was significantly enhanced in the presence of two cSP homologs (cSPHs), cSPH11 and cSPH50, suggesting the latter are cofactors for PPO activation. Moreover, baculovirus infection was efficiently blocked by the reconstituted PPO activation cascade, and the effect was boosted by cSPH11 and cSPH50. Taken together, we unraveled a conserved PPO activation cascade in *H. armigera*, which is similar to that exists in lepidopteran biochemical model *Manduca sexta* and highlighted its role in antagonizing viral infection.

## Introduction

Melanization is a prominent defense mechanism in arthropods that plays an essential role in wound healing, killing of microbes, and parasites encapsulation (1, 2). The key protease in melanization is phenoloxidase (PO), which can catalyze phenols to quinines, then form melanin. PO usually exists as the zymogen, prophenoloxidase (PPO). Its activation depends on the extracellular serine protease (SP) cascade triggered by invading microbes. The recognition of pathogen-associated molecular patterns, such as β-1,3-glucan from fungi, peptidoglycan from Gram-positive bacteria, or lipopolysaccharide from Gram-negative bacteria, by host pattern recognition receptors (PRRs) leads to the activation of modular proteases that sequentially cleave the downstream SPs and ultimately activate PPO (3).

The extracellular PPO activation pathway usually consists of a three-step proteolytic cascade initiated by one modular SP then followed by clip domain SPs (cSPs), which has been comprehensively revealed in a lepidopteran species *Manduca sexta* (4–7) and a coleopteran species *Tenebrio molitor* (8, 9). cSPs and the homologs are classified into four subfamilies (A–D) based on phylogenetic analysis (10, 11). Most PPO activating proteases that directly activate PPO belong to CLIPB, such as *M. sexta* PPO activating protease (PAP) 1-3 (12, 13) and *T. molitor* Spätzle processing enzyme (SPE) (8). The proteases that cleave CLIPB are generally derived from CLIPC. For example, *M. sexta* hemolymph protease (HP) 6 and HP21 activates PAP1 and PAP2/3, respectively (4, 7) and *T. molitor* SPE activating enzyme (SAE) cleaves SPE (8). The initiating modular SPs without clip domains that activate CLIPC members are characterized by containing low-density lipoprotein receptor class A (LDLa), Sushi and Wonton domains (14, 15). They could be autoactivated in the presence of pathogens, then cleaved the downstream proteases. In *M. sexta*, the modular SP, HP14, was stimulated to activate by its interaction with β-glucan recognition proteins (βGRP) 2 before cleaving HP21 (15). *T. molitor* modular SP (MSP) was also one modular SP which activated SAE (8). Alternatively, the initiating SP could be the CLIPD member. For example, *M. sexta* HP1, a member of CLIPD, was identified as a recognition protein of the melanization cascade which was activated without proteolytic cleavage (3, 16).

CLIPA are cSP homologs (cSPHs) that lost catalytic activity due to the replacement of catalytic triad residues (11). cSPHs seem to serve as cofactors that significantly increase PO activity (6, 12, 13). Although there were three PAPs in *M. sexta*, PO activity was very low in the absence of cofactors. Only in the presence of cSPH1 and cSPH2, PO activity was greatly enhanced (12). According to the crystal structure of *M. sexta* PPO, it has been suggested that the combination of cSPHs and PO might lead to the conformation change of the latter, enabling the substrate to be more accessible to the active site of PO (17).

Melanization has also been studied in other insects. In *Drosophila melanogaster*, Hayan, Sp7 and ModSP were verified to function during melanization (18, 19). In *Aedes aegypti*, immune melanization proteases (IMP-1 and IMP-2) were identifed to mediate the cleavage of PPO to combate the malaria parasite (20). In *Anopheles gambiae*, CLIPB9 directly cleaves and activates PPO, whereas CLIPB8 is also part of the PPO activation system (21, 22). In *Bombyx mori*, PGRP-S5 functions as a pattern recognition receptor during melanization (23) and BmSPH-1 interacts with PPO and PPO-activating enzyme (PPAE) (24). In *Ostrinia furnacalis*, SP105 could fucntionaly activate PPO (25). Overall, researches on melanization in other insects are not as comprehensive as those in *M. sexta* and *T. molitor*.

Several studies have suggested that melanization is involved in defense against virus infection. For examples, silencing PPO-I gene in *Armigeres subalbatus* increased Sindbis virus replication (26). Plasma PO of *Heliothis virescens* inhibited baculovirus infection (27). The melanin precursor 5,6-dihydroxyindole (DHI) showed broad-spectrum antiviral activity (28). PO activity in *Ae. aegypti* is required for innate immune response against Semliki Forest virus (SFV) infection (29). Recently, our study showed that melanization in *Helicoverpa armigera* is involved in baculovirus infection (30).

Cotton bollworm, *H. armigera*, is a worldwide distributed agricultural pest. It caused severe damage to many crops (31). Melanization in *H. armigera* plays an important role in defense against invading pathogens (30, 32–35). Previously transcriptomic and proteomic analyses showed that many SPs and homologs were up-regulated in response to the challenge of bacteria or fungi (34), however, they were down-regulated with baculovirus infection (30). At the same time, two negative regulators serpin-5 and serpin-9 of the pathway were sequentially induced by baculovirus infection to inhibit their target proteases, cSP4 and cSP6, respectively (30). Thus, baculoviruses have developed efficient strategies to suppress the host melanization response for their proper proliferation. Previous studies identified that there were two PPOs (PPO1 and PPO2) and at least 11 cSPs in *H. armigera* (34). These include procSP6, 7, and 8 belonging to CLIPB; procSP1, 2, 3, and 4 of CLIPC; and procSP5, 9, 10, and 29 belonging to CLIPD. In addition, three potential mudular SPs (proSP41, 42, and 43) were identified with the LDLa and sushi domains, while procSPH11, 49, and 50 were found to be cSP homologs. Furthermore, it has been verified that PPO can be proteolytically activated by cSP6, a member of the CLIPB subfamily (30). However, so far, the complete PPO activation pathway of *H. armigera* remains unclear.

In this study, we identified the members involved in PPO activation cascade step-by-step using biochemical methods and finally *in vitro* reconstructed a complete PPO activation pathway in *H. armigera*. Two cSPHs that could significantly enhanced PO activity were identified. The reconstructed PPO activation pathway efficiently antagonized viral infection *in vitro*. The cascade in *H. armigera* was conserved compared with that in *M. sexta*.

## Materials and Methods

### Cells and Virus

The *Drosophila* S2 cell line was cultured in ESF921 medium (Expression Systems, Woodland, CA, United States) at 27°C. The recombinant *Helicoverpa armigera* nucleopolyhedrovirus (HearNPV) expressing an *egfp* reporter gene (HearNPV-*egfp*) was previously constructed by our laboratory (36).

### Expression of Recombinant Serine Proteases (SPs)

Total RNA was isolated from the fat body of the day-3 5th instar *H. armigera* larvae using TRIzol reagent (Invitrogen, Carlsbad, CA, United States). The entire coding region of proSPs (proSP41, procSP1, procSP6) and procSPHs (procSPH11, procSPH49 and procSPH50) (34) were amplified by reverse transcriptase polymerase chain reaction (RT-PCR) using the PrimeScript^TM^ RT reagent kit with gDNA Eraser (Takara Bio, Otsu, Japan) with the primers listed in Supplementary Table S1. The PCR products were cloned into the pMT-BiP/V5-HisA vector (Invitrogen). Overlap extension PCR was performed to prepare constructs designated as cSP_Xa_, in which four residues at the putative activation site were replaced with tetrapeptide IEGR, a cleavage site of bovine coagulation factor Xa (37). The putative cleavage sites of proSP41, procSP1, procSP6, procSPH11, procSPH49 and procSPH50 are VDVL, TDKL, VGNK, ADLR, VSFI, and LDIR, respectively. The plasmids were transfected into *Drosophila* S2 cells along with pCoHygro hygromycin selection vector (Invitrogen) and stable cell lines were screened according to the manufacturer’s instruction. The cell supernatants containing secreted recombinant proteases were harvested. Recombinant proteins were purified using nickel-charged resin (Roche Diagnostics, Basel, Switzerland), eluted with imidazole, and further concentrated by filtration through an Amicon Ultra 10K cartridge (Millipore, Billerica, MA, United States). The purified proteins were stored at −80°C before use.

### Generation of Polyclonal Antibodies

procSP6, procSPH11, and procSPH50 for prokaryotic expression were subcloned into the pET-28a expression vector using the primers listed in Supplementary Table S1. Recombinant protein was expressed in *Escherichia coli* BL21 cells and purified with nickel-charged resin. procSP1 was expressed in *Drosophila* S2 cells as described above. The recombinant proteins were used to immune rabbit to generate the respective polyclonal antibodies as described previously (38). The polyclonal antibodies against PPO1 and PPO2 were generated as described previously (30).

### Purification of PPO From Larval Hemolymph

Prophenoloxidase was purified from the hemolymph of day-3 5th instar *H. armigera* larvae according to the protocol reported described (30). Briefly, 10 ml hemolymph was collected from larval body and pooled into ice-cold saturated ammonium sulfate (AS). AS saturation (35-50%) was collected and loaded on column prepacked with Ceramic Hydroxyapatite (Bio-Rad, Hercules, CA, United States). The fractions with cetylpyridinium chloride (CPC) activated PO activity were combined and applied through Concanavalin A Sepharose column (Sigma-Aldrich, St. Louis, MO, United States). The flow-through fraction was applied to a Phenyl Sepharose 6 Fast Flow (low sub) column (GE Healthcare, Little Chalfont, United Kingdom). Fractions containing PO activity were applied to a Superdex 200 column (ÄKTApurifier; GE Healthcare). Purified PPO were stored at −80°C before analysis.

### The Activation and Activity of Serine Protease and PPO

To activate procSP_Xa_ with factor Xa, purified procSP_Xa_ was incubated with bovine factor Xa (New England Biolabs, Ipswich, MA, United States) in buffer [20 mM Tris–HCl, 0.1 M NaCl, 2 mM CaCl (pH 8.0)] at 27°C for 5 h. Amidase activity of the reaction mixtures was measured using 200 μL, 50 μM acetyl-Ile-Glu-Ala-Arg-p-nitroanilide (IEAR) as the substrate (39). One unit of amidase activity was defined as ΔA405 of 0.001 in one minute. Factor Xa activated procSP_Xa_ was incubated with procSP at 37°C for 1 h before immunoblot analysis under reducing conditions containing β-mercaptoethanol (β-ME) or non-reducing conditions. Mixtures containing sequentially activated SP cascade components (cSP6_Xa_, cSP1_Xa_/procSP6, and SP41_Xa_/procSP1/procSP6) were incubated with purified PPO at room temperature for 10 min to detect PPO cleavage by immunoblotting. To measure PO activity, samples were transferred to 96-well plates, and 200 μL of 2 mM Dopa in 50 mM sodium phosphate buffer (pH 6.5) were added. The activity was determined by measuring the absorbance at 470 nm with a microplate reader (Synergy H1; BioTek, Winooski, VT, United States). One unit of PO activity was defined as ΔA470 of 0.001 in one minute (30).

### Effects of *in vitro* Activated Melanization on Baculovirus Infection

HearNPV-*egfp* (MOI = 0.5 TCID_50_ units/well) was mixed with the SP cascade (SP41_Xa_ + procSP1 + procSP6), PPO and its substrate (PPO + Dopa), the cSPHs (procSPH11 + procSPH50), and the serine protease inhibitor (serpin-9) with different combinations. The amount of each agents were as follows: 200 ng PPO, 10 μL of 20 mM Dopa, 50 ng SP41_Xa_, 50 ng procSP1, 100 ng procSP6, 200 ng procSPH11, 200 ng procSPH50, and 1 μg serpin-9. Then all of the mixtures were adjusted to a final volume of 100 μL and incubated at room temperature for 0, 1, and 3 h, respectively. The mixtures were added to HzAM1 cells in Grace’s insect medium supplemented with 2% fetal bovine serum in 24-well plates and incubated for 2 h. The cells were washed three times with serum-free medium and incubated at 27°C for 24 h, and viral infection was examined under a fluorescence microscope using the EVOSTM FL Auto Imagine System (Thermo Fisher Scientific, Waltham, MA, United States).

### Statistical Analysis

All statistical evaluations were determined using GraphPad Prism 5 software. Statistical differences between two groups were performed using the two-tailed Student’s *t*-tests (*n* ≥ 3 biological replicates) ^∗^*p* < 0.05, ^∗∗^*p* < 0.01, and ^∗∗∗^*p* < 0.001.

### Gene Accession Numbers

All sequence data that support the findings of this study are available in GenBank with the following accession numbers: proSP41 (MT182806), proSP42 (MT182807), proSP43 (MT182808), procSP1 (MT182805), procSP6 (KY680241), procSPH11 (MT182809), procSPH50 (MT182810), PPO1 (KY744277), PPO2 (KY744278), and serpin-9 (KY680239).

## Results

### cSP1 Cleaves the PPO Activating Protease cSP6

We decided to *in vitro* re-constitute the PPO activation cascade of *H. armigera* using a “bottom-up” strategy. PPO was purified from the hemolymph of *H. armigera* larvae and, after a CPC-induced conformation changes, PO activity was confirmed by production of dopamine chrome (or dopachrome) from dopamine (or dopa) (Supplementary Figure S1A). Immunoblotting analysis further showed that purified PPO formed a heterodimer constituted of PPO1 and PPO2 (Supplementary Figure S1B). We previously identified that cSP6 served as a PPO activating enzyme (30). This was confirmed as evidenced by the cleavage and enzymatic activation of PPO by the factor Xa activated recombinant procSP6_Xa_ (Supplementary Figures S1C,D).

**FIGURE 1:**
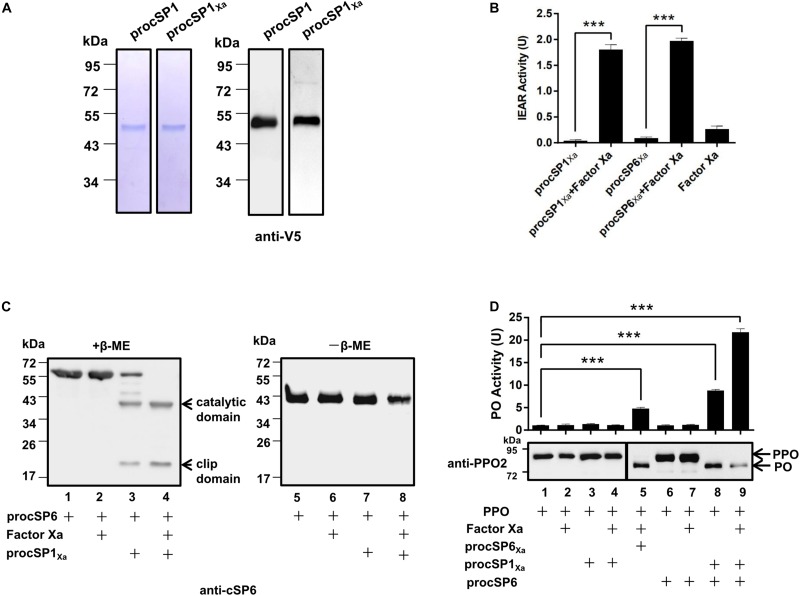
Prophenoloxidase (PPO) is sequentially activated by cSP1/cSP6. **(A)** SDS-PAGE and immunoblot analysis of purified recombinant procSP1 and procSP1_Xa_. Anti-V5 antibody was used to detect recombinant proteins by immunoblotting. **(B)** Amidase activity of cSP1_Xa_. Catalytic activity of activated cSP1_Xa_ (100 ng) and cSP6_Xa_ (100 ng) were detected using IEAR as a substrate. ***p < 0.001. **(C)** Factor Xa activated procSP1_Xa_ can cleave procSP6. procSP1_Xa_ (50 ng) was processed by factor Xa, and then incubated with procSP6 (100 ng) for 1 h. To examine the effect of disulfide bonds on protein mobility, mixtures were treated with SDS sample buffer with (left panel) or without β-ME (right panel) and separated by SDS-PAGE followed by immunoblotting using an anti-cSP6 antibody. **(D)** PPO was sequentially activated by cSP1/cSP6. Activated cSP6 was incubated with PPO (100 ng) for 10 min, and analyzed by immunoblotting using an anti-PPO2 antibody (middle panel). Higher amount of PPO (300 ng) was used in detecting PO activity (upper panel), and PO activity was represented as mean ± SD of three independent experiments. ***p < 0.001.

According to the phylogenetic analysis, cSP1 of *H. armigera* was classified as a member of CLIPC subfamily, and showed close phylogenetic relationship to *M. sexta* HP21 (30), the upstream cSP of *M. sexta* PAP2/3 (7), implying that cSP1 might be the protease upstream of cSP6 in *H. armigera*. To characterize the function of cSP1, recombinant procSP1 and its modified form were expressed and purified using *Drosophila* S2 cells (Figure 1A). Activity of the cleaved cSP1 and cSP6 was detected as hydrolysis of the IEAR substrate (Figure 1B). Then, procSP6 was incubated with factor Xa activated procSP1_Xa_, and the result showed that cSP1_Xa_ could cleave procSP6 (∼57 kDa), and the separated catalytic domain (∼38 kDa) and clip domain (∼19 kDa) were clearly detected with the anti-cSP6 antibody under reducing condition (Figure 1C, lane 4). Interestingly, procSP6 could be partially cleaved by procSP1_Xa_ without activation (Figure 1C, lane 3). While under the non-reducing condition, the disulfide bond linked subdomains of cSP6 migrated to the same position as the procSP6 (Figure 1C, lanes 5–8), indicating that procSP6 was specifically cleaved by cSP1_Xa_.

Next, PPO was added to the mixtures as described above and the cleavage of PPO was detected using immunoblotting. As expected, PPO was efficiently cleaved by cSP6 in the presence of procSP1_Xa_ and factor Xa (Figure 1D, lane 9). Correspondingly, high PO activity was detected (Figure 1D, lane 9, upper panel). Interestingly, procSP1_Xa_ and procSP6 mixed together were able to activate PPO in the absence of factor Xa (Figure 1D, lane 8), which was consistent with the finding that procSP6 was partially cleaved by procSP1_Xa_ (Figure 1C, lane 3). We noticed that PO activity induced by cSP6 via activated cSP1 (Figure 1D, lane 9) was much higher than that by factor Xa activated cSP6_Xa_ (Figure 1D, lane 5), indicating that cSP6 activated at its native cleavage site has higher activity than the modified form. To be noted, PO activity induced by cSP6 via procSP1_Xa_ (Figure 1D, lane 8) was also higher than that by factor Xa activated cSP6_Xa_ (Figure 1D, lane 5), suggesting the self-activated procSP1_Xa_ is likely to be able to active cSP6 at its native cleavage site. Thus, PPO can be activated by the cascade of cSP1/cSP6.

### SP41 Is an Initiating SP of the PPO Activation Pathway

**FIGURE 2:**
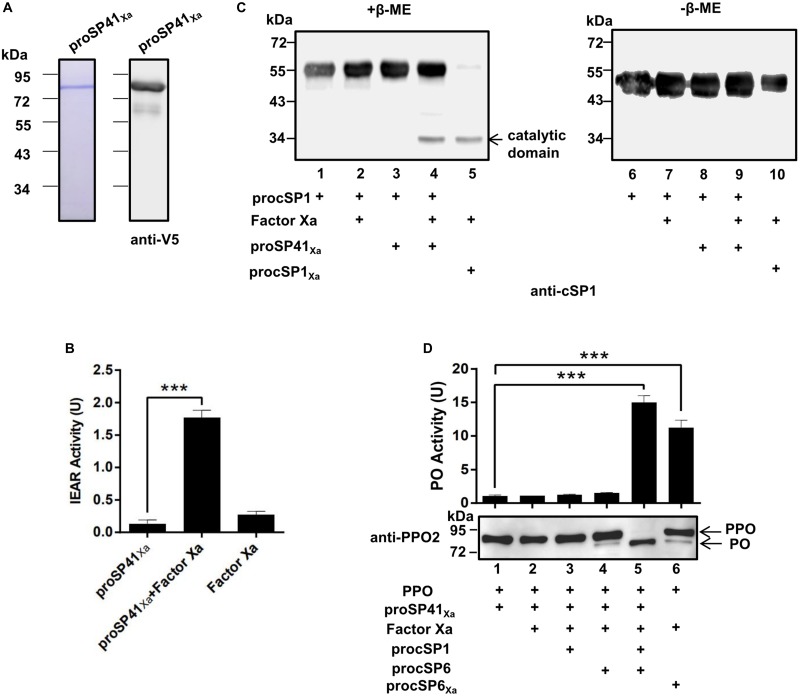
Prophenoloxidase activation by the melanization cascade initiated by SP41. **(A)** SDS-PAGE and immunoblot analysis of purified recombinant proSP41 and proSP41_Xa_. Anti-V5 antibody was used to detect recombinant proteins by immunoblotting. **(B)** Amidase activity of SP41_Xa_. Catalytic activity of activated SP41_Xa_ (300 ng) was detected using IEAR as a substrate. ***p < 0.001. **(C)** Activation of procSP1 by SP41_Xa_. Factor Xa (50 ng) activated proSP41_Xa_ was incubated with procSP1 (100 ng) for 1 h. To examine the effects of disulfide bonds on protein mobility, mixtures were treated with SDS sample buffer with (left panel) or without β-ME (right panel) and analyzed by immunoblotting using an anti-cSP1 antibody. **(D)** PPO activation by the melanization cascade initiated by SP41_Xa_. Activated cSP1 in **(C)** was incubated with procSP6 (50 ng) for 1 h, and then mixed with 100 ng PPO with immunoblotting or 300 ng PPO with PO activity for another 10 min. Immunoblotting was performed using an anti-PPO2 antibody (middle panel). PO activity (upper panel) was represented as mean ± SD of three independent experiments. ***p < 0.001.

To find out the initiating SP in the PPO activation pathway of *H. armigera*, phylogenetic analysis (Supplementary Figure S2A) and domain architecture comparison (Supplementary Figure S2B) were performed. Three modular SPs (SP41, SP42, and SP43) in *H. armigera* showed homology (with the identities of 48, 58, and 44%, respectively) to *M. sexta* HP14, which is an initiating SP upstream of HP21 (14, 15), implying the possible role of the three SPs in activation of procSP1. To verify their functions, recombinant proSP41_Xa_, proSP42_Xa_, and proSP43_Xa_ were expressed and purified using *Drosophila* S2 cells (Supplementary Figures 2A, S3A). The SP activity was measured using IEAR substrate, and the result showed that purified recombinant modular SP41_Xa_ exhibited amidase activity (Figure 2B), so did SP42_Xa_ and SP43_Xa_ (Supplementary Figure S3B). Then proSP41_Xa_, proSP42_Xa_, and proSP43_Xa_ were tested for their ability to cleave procSP1. Among the three cSPs, only SP41_Xa_ cleaved procSP1 (Figure 2C, lane 4) and the catalytic domains of cSP1 migrated to the same position with procSP1 under non-reducing conditions, indicating that it was specifically cleaved (Figure 2C, lanes 6–10). In contrast, proSP42_Xa_ and proSP43_Xa_ failed to activate procSP1 (Supplementary Figure S3C).

We next investigated whether the entire pathway could activate PPO *in vitro*. The PO band was clearly detected after incubation of PPO with the mixtures of factor Xa, proSP41_Xa_, procSP1, procSP6 (Figure 2D, lane 5), and PO activity was also increased (Figure 2D, lanes 5 and 6, upper panel). These results clearly showed that PPOs were enzymatically cleaved and activated by the cascade initiated from activated SP41_Xa_. Thus, a complete PPO activation pathway in *H. armigera* was reconstructed *in vitro*.

### PO Activity Is Enhanced in the Presence of cSPH11 and cSPH50

**FIGURE 3:**
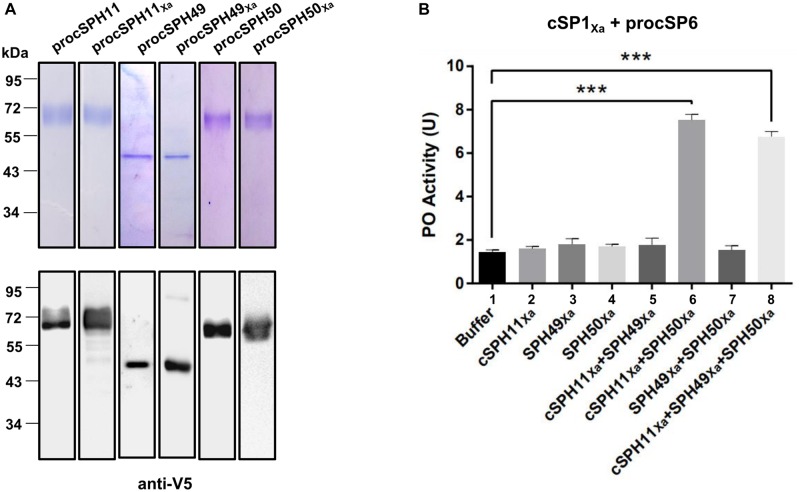
Phenoloxidase (PO) activity is enhanced by cSPH11_Xa_ and cSPH50_Xa_. **(A)** SDS-PAGE and immunoblot analysis of recombinant procSPH11, procSPH11_Xa_, procSPH49, procSPH49_Xa_, procSPH50, and procSPH50_Xa_. Anti-V5 antibody was used in immunoblotting. **(B)** PO activity increased significantly in the presence of cSPH11_Xa_ and cSPH50_Xa_. Factor Xa processed procSP1_Xa_ (50 ng) was incubated with procSP6 (100 ng) for 1 h, respectively. At the same time, procSPH11_Xa_, procSPH50_Xa_ and procSPH49_Xa_ (100 ng) were activated with factor Xa. Purified PPO (100 ng) was added to the mixture and PO activity was measured. PO activity was represented as mean ± SD of three independent experiments. ***p < 0.001.

Phylogenetic analysis showed that three *H. armigera* cSPHs (cSPH11, cSPH49, and cSPH50) were homologs to *M. sexta* cSPH1 and cSPH2 (data not shown), suggesting that they may serve as potential cofactors for PPO activation. Therefore, we firstly expressed and purified recombinant procSPHs and their modified forms (Figure 3A). Then, the factor Xa activated procSPHs, either individually or in different combinations, were incubated with mixtures of PPO and cSP1_Xa_ activated cSP6 before measuring of PO activity. Only in the presence of cSPH11_Xa_ and cSPH50_Xa_ simultaneously, a significant increase of PO activity was detected (Figure 3B, lane 6 and 8), indicating that cSPH11 and cSPH50 acted in concert to synergize PO activity. To be noted, to better reflect the function of cSPHs, the amount of PPO used in this experiment (Figure 3B) was much lower than the above results when cSPHs were not present (Figures 1D, 2D).

**FIGURE 4:**
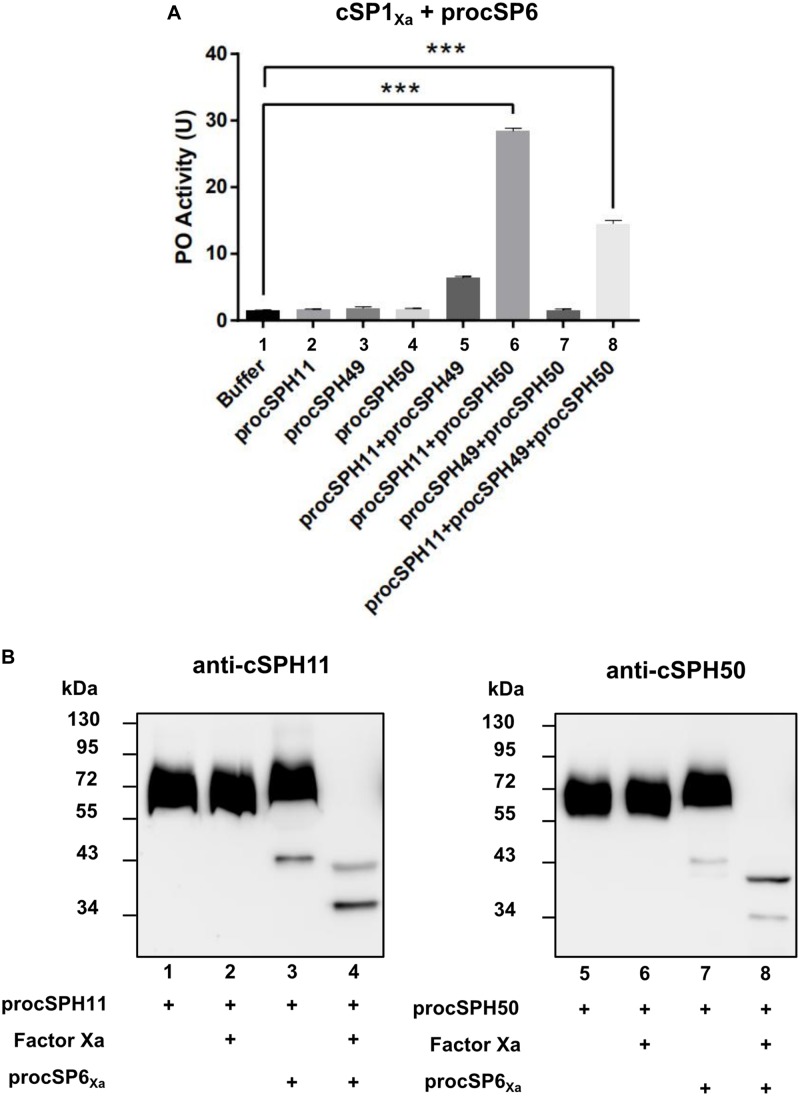
cSPH11 and cSPH50 are cofactors in PPO activation. **(A)** PO activity was increased significantly in the presence of procSPH11 and procSPH50. The experimental groups were the same with Figure 3B, instead of factor Xa mutants, wild type procSPHs were used to measure PO activity (upper panel), which was represented as mean ± SD of three independent experiments. ***p < 0.001. **(B)** Proteolytic activation of procSPH11 and proSPH50 by cSP6_Xa_. Factor Xa activated cSP6_Xa_ (50 ng) was incubated with 100 ng procSPH11 (left panel) or procSPH50 (right panel) for 1 h. The mixtures were analyzed by immunoblotting using anti-cSPH11 or anti-cSPH50 antibody.

To further confirm this finding, we performed a similar experiment as Figure 3B by using purified wild type forms of procSPH11, procSPH49 and procSPH50 instead of the modified procSPHs activated with factor Xa. The result showed that PO activity was also increased in the presence of procSPH11 and procSPH50 (Figure 4A, lane 6), with even much higher activity (about fourfold greater) than those using the factor Xa activated cSPHs. Interestingly, the combination of cSPH11 and cSPH49 also increased PO activity (Figure 4A, lane 5) but the effect was less prominent than that induced by cSPH11 and cSPH50 (Figure 4A, lane 6).

In *M. sexta*, cSPHs could be cleaved by PAPs, which were PPO activating proteases (12). Therefore, we asked whether cSPHs would be cleaved by the PPO activating protease before functioning in *H. armigera*. To examine this hypothesis, factor Xa activated cSP6_Xa_ was incubated with procSPH11 (Figure 4B, left panel) or procSPH50 (Figure 4B, right panel) at 37°C for 1 h, and then analyzed using immunoblotting. As expected, cleaved bands corresponding to cSPH11 or cSPH50 were detected, when cSP6_Xa_ was activated (Figure 4B, lanes 4 and 8).

### *In vitro* PPO Activation Cascade Blocks Baculovirus Infection

**FIGURE 5:**
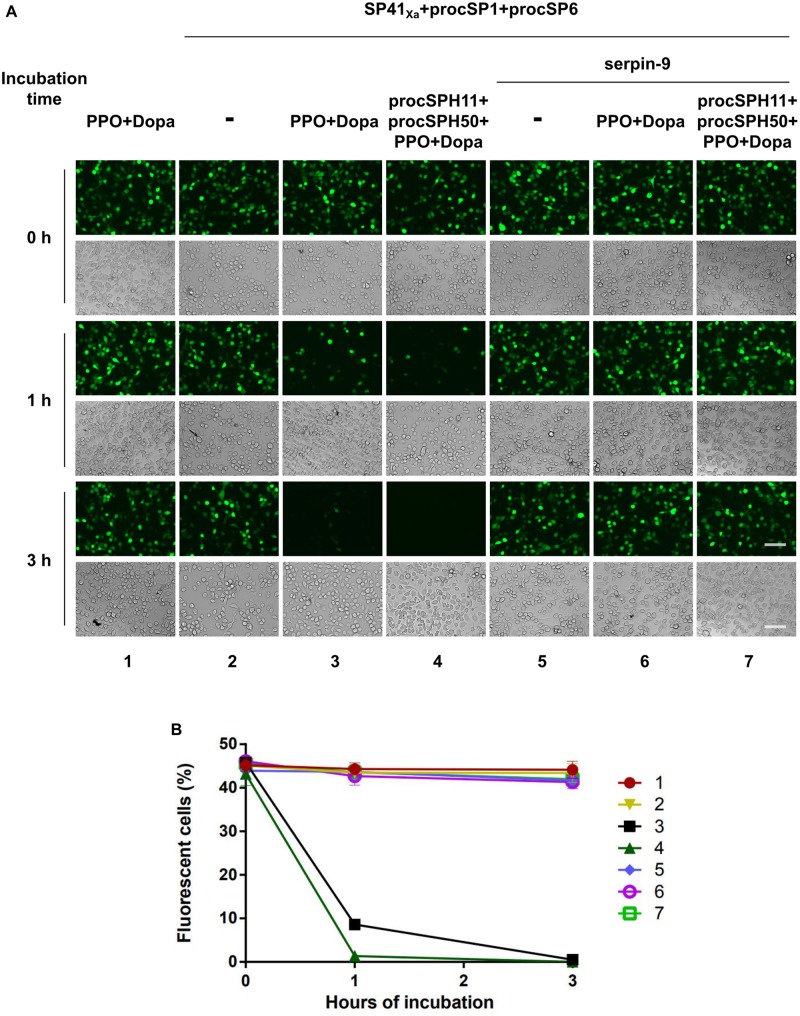
Baculovirus infection is blocked by melanization cascade *in vitro*. **(A)** HearNPV-*egfp* was mixed with PPO and Dopa, SP cascade (SP41_Xa_ + procSP1 + procSP6), the cSPHs (procSPH11 + procSPH50), and the serine protease inhibitor (serpin-9) with different combinations. These mixtures were incubated at room temperature for 0, 1, or 3 h before infecting HzAM1 cells. Fluorescence micrographs and normal imagines were acquired 24 h p.i. to assess the viability of the baculovirus. Scale bars represent 100 μm. **(B)** Quantification of fluorescent cells. Infected HzAM1 cells in images shown in **(A)** were counted. All data were represented as mean ± SD of three independent experiments. Labels 1–7 in the figure represented different treatments as indicated in **(A)**.

Melanized hemolymph of *H. armigera* could inactivate the infectivity of HearNPV in cell cultures (30). Since hemolymph consists of complicated components, we would like to evaluate whether our identified melanization cascade could directly block viral infection when activated *in vitro*. To this end, an *egfp* maker gene labeled HearNPV-*egfp* was incubated with purified PPO, the substrate Dopa, and selected cSP or SP for 0, 1, and 3 h at room temperature. Then, the mixtures were added to HzAM1 cells for 24 h before observation using fluorescence microscopy. When the HearNPV-*egfp* suspension was incubated with the mixtures of PPO and Dopa, the number of infected cells was similar among the 0, 1, and 3 h incubation groups (Figure 5, panel 1), suggesting that inactivated PPO and the substrate did not affect virus viability. Similarly, when the cascade components (SP41_Xa_, procSP1, and procSP6) were incubated with the virus, respectively, the number of infected cells was similar at 0, 1, and 3 h post infection (h p.i.) (Figure 5, panel 2), indicating that these proteins alone had no effects on virus infectivity. However, in the presence of PPO and Dopa in addition to the cSP6 cascade components, much fewer infected cells were observed after 1 h, and almost no virus infection was observed after 3 h (Figure 5, panel 3), demonstrating that the cSP6 mediated PPO activation could block viral infection efficiently. When procSPH11 and procSPH50 were added to the above mixtures, a more potent inhibitory effect was observed after 1 h p.i., and no virus infected cells were detected (Figure 5, panel 4). Furthermore, when serpin-9, an inhibitor of cSP6 (30), was added to the mixtures, viral infection was substantially rescued (Figure 5, panel 6 and 7). These results demonstrated that the SP41/cSP1/cSP6 cascade can induce melanization and block baculovirus infection. Moreover, the inhibitory effect against baculovirus infection was enhanced in the presence of the cofactors and the inhibition could be rescued by serpin-9.

## Discussion

**FIGURE 6:**
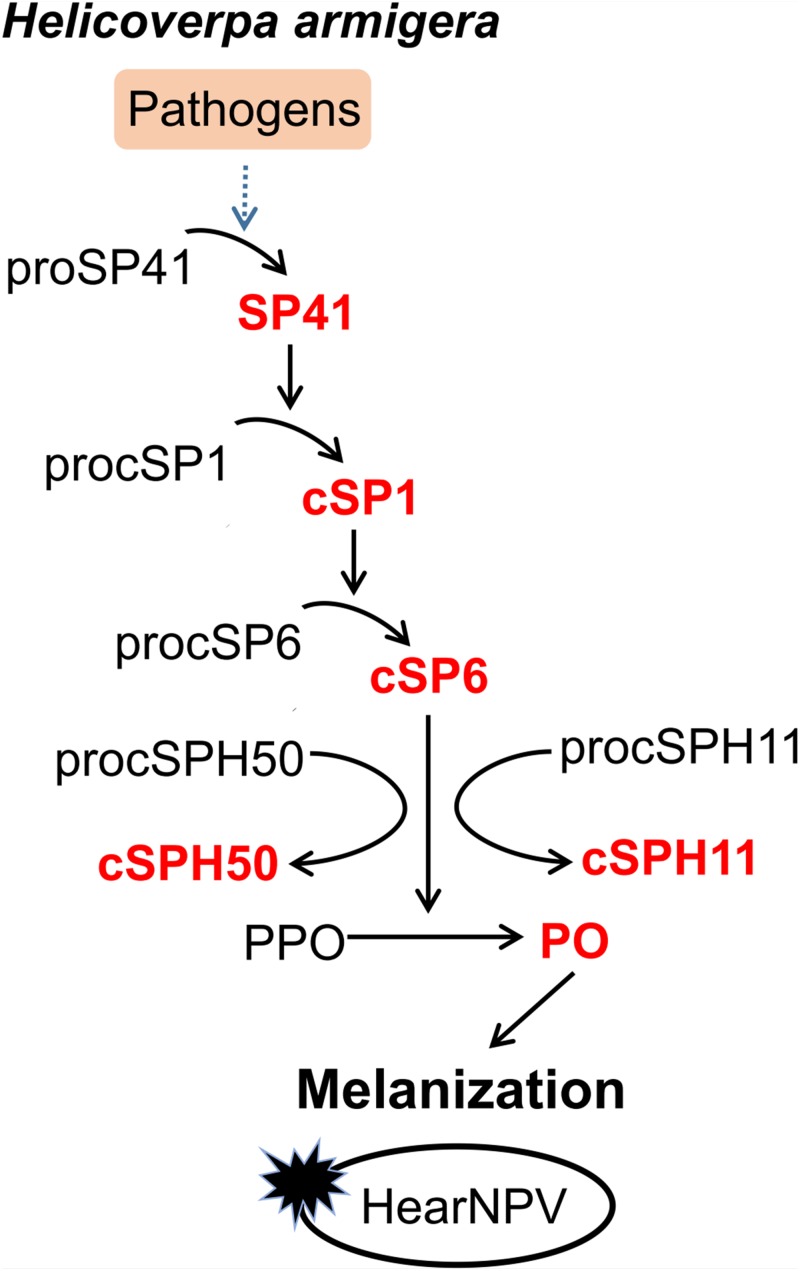
Proposed PPO activation pathway in *H. armigera*. Initiation of SP41 sequentially cleaves cSP1/cSP6, resulting in PPO activation. The cofactors cSPH11 and cSPH50 can enhance PO activity activated by cSP6. Baculovirus infection can be blocked by melanization *in vitro*.

Although certain components of melanization cascade have been identified in many insects, such as *Ae. aegypti* (10, 20, 40, 41), *A. gambiae* (42–45), *D. melanogaster* (18, 46, 47), the complete PPO activation pathway was elucidated only in a few insects, for example *M. sexta* (4–7), and *T. molitor* (8, 9). To date, the complete pathway in *H. armigera* was unknown until this study. Transcriptome-based analysis revealed more than 60 SPs and homologs in *H. armigera*. Among these, at least 11 clip domain-containing members might be involved in PPO activation cascades (34). However, only cSP4 and cSP6 were confirmed to participate in *H. armigera* PPO activation pathway (30). Here, based on the PPO activating protease activity of cSP6, a PPO activation pathway composed of its activating protease cSP1 and the initiating protease SP41 was identified and reconstituted *in vitro* using biochemical methods. In addition, cSPH11 and cSPH50, which could be cleaved by the terminal cSP6, were characterized as the cofactors during PPO activation. The PPO pathway identified in *H. armigera* (Figure 6) resembles the HP14/HP21/PAP2/3 pathway of *M. sexta* (48, 49).

The initiating proteases of melanization are generally autoactivated upon binding of PRRs to pathogens (14, 15, 47, 49, 50). For *M. sexta* HP14, binding of β-1,3-glucan to βGRP2 results in a significant increase in affinity between the N-terminal LDLa domains of HP14 and βGRP2 (15). MSPs in other insects such as *D. melanogaster* (47) and *T. molitor* (8) are considered as initiating proteases in SP cascades and they also contain LDLa domains. Similarly, the three SPs (SP41-43) of *H. armigera* all have LDLa domains (Supplementary Figure S2B), however, only SP41 was able to induce melanization cascade (Figure 2 and Supplementary Figure S3). In the domain structure, both SP41 and SP42 contain five LDLa domains and two Sushi domains at their N-termini, while SP43 has only four LDLa domains (Supplementary Figure S2B). Currently it is unclear why only SP41, but not the other two SPs serve as the initiating SP. Besides the modular SPs, cSPs may also function as the initiating SPs. *M. sexta* proHP1 utilizes a conventional mechanism to active its downstream protease which was not induced by proteolytic cleavage (16). Whether there exists another PPO activation cascade in *H. armigera* initiated by a clip domain SP remains to be determined.

Various mechanisms of PPO activation by the terminal cSPs and cofactors have been characterized in insects (2). In *B. mori*, PPO1 and PPO2 are cleaved by PPAE belonging to CLIPB, and the resulting large fragments of PO1 and PO2 directly exhibit PO activity (51). In *M. sexta*, cleavage of PPO1 and PPO2 by three PAPs (CLIPB) yielded large fragments of PPO1 and PPO2 with low PO activity, which was significantly enhanced by the SPH1 and SPH2 (CLIPA). During this process, SPHs must be cleaved by PAPs then to play their roles (6, 12, 13). In *Holotrichia diomphalia*, PPO-activating factor (PPAF)-I is a CLIPB protease which cleaves PPO-I to generate a 76 kDa fragment without PO activity; however, when PPAF-II (CLIPA) and PPAF-III (CLIPB) were further added, a new 60 kDa fragment with PO activity was produced (52). The crystal structure of PPAF-II showed that its clip domain adopted a novel conformation compared to CLIPB members then may serve as a module for binding the cleaved PO and forming active PO clusters (53). How PPAF-I, II and III act in concert to activate PPO remains to be determined. In *Ae. aegypti.* ten PPO genes were identified and a 50 kDa PO fragment was generated challenged by fungi, suggesting a complicated activation mechanism (40). In *H. armigera*, our results showed that the cofactors procSPH11 and procSPH50 were also cleaved by cSP6 (Figure 4B). There was low PO activity after PPO was cleaved by cSP6, and PO activity was significantly increased in the presence of cSPH11 and cSPH50 which are orthologs of *M. sexta* SPH1 and SPH2, respectively. Our results suggested that the mode of PPO activation in *H. armigera* was similar to that in *M. sexta*. It will be interesting to elucidate the mode of actions of the cofactors in insect melanization responses in the future.

Melanization is essential for combating pathogens in insects. In *Ae. aegypti*, PO activity was found to be required for defense against the SFV (29). Knocking down the only two PPO genes of *Penaeus monodon* led to the increased mortality by white spot syndrome virus (WSSV) infection (54). These suggest that melanization plays a crucial role in antiviral immunity. Correspondingly, viruses have evolved versatile strategies to inhibit or escape host melanization response for their proper survival, either by inhibiting the signal transduction of melanization or affecting PO activity directly. The polydnaviruses (PDV) carried by the *Microplitis demolitor* expresses Egf1.0 and Egf1.5 to inhibit the activity of PAP1 and PAP3 of *M. sexta* (55, 56). Infection of *Ae. aegypti* with Egf1.0-expressing SFV led to increased mortality and virus amplification (29). WSSV453, a non-structural viral protein, interacts with *P. monodon* proPPAE2 and interferes with its activation to active PPAE2 (57). In *H. armigera*, a transcriptomic analysis showed that cSP6 was markedly repressed during the late stage of baculovirus infection, and the inhibitor of cSP6 was up-regulated to suppress melanization (35). Although a previous study demonstrated that the melanized hemolymph of *H. armigera* could inactive virus (30), considering the complexity of hemolymph components, there might be other antiviral host factors involved in. Through the reconstruction of melanization *in vitro*, we demonstrated that activated melanization reaction itself could inhibit baculovirus infectivity (Figure 5). Thus, melanization response in *H. armigera* was confirmed to play an important and direct role in combating baculovirus infection. Baculovirus has a bilateral life cycle that it uses occlusion derived viruses (ODVs) to initiate midgut infection and budded viruses (BVs) for systemic infection. As melanization happens in hemolymph and it inactived BV infection *in vitro* (Figure 5), we propose melanization prevents systemic infection of baculovirus by inactivating BVs in the infected hemolymph.

Recently, a third PPO pathway comprising HP14/HP2/PAP2 was identified in *M. sexta*, largely activated in wandering larvae and pupae (58). Considering that there are two PPO activating proteases (cSP6 and cSP8) in *H. armigera* (30), it is possible that there might be at least two branches of melanization pathways in this species. The multiple melanization cascades may be involved in specific recognition of different pathogens and may provide a more complete protection of insects in combating against invading pathogens. Further efforts are required to characterize the complete melanization pathways/network in *H. armigera*. In addition, how virus interacts with PRRs or initiating SPs of PPO activation cascade is also worth further exploration. Taken together, our findings provide an important first step toward understanding the complicated melanization network in *H. armigera*.

## Data Availability Statement

All datasets generated for this study are included in the article/Supplementary Material.

## Author Contributions

QW, ZH, ZZ, and MW designed the experiments, interpreted the data, and wrote the manuscript. QW, MY, CY, and XL assisted with the experiments and provided critical reagents and intellectual input. ZH, ZZ, and MW supervised the study.

## Conflict of Interest

The authors declare that the research was conducted in the absence of any commercial or financial relationships that could be construed as a potential conflict of interest.

## Abbreviations

β GRP: β -glucan recognition proteins
β -ME: β -mercaptoethanol
AS: ammonium sulfate
CPC: cetylpyridinium chloride
cSP: clip domain serine protease
cSPH: clip-domain serine protease homolog
cSPH: cSP homologs
HearNPV: *Helicoverpa armigera* nucleopolyhedrovirus
HP: hemolymph protease
IEAR: acetyl-Ile-Glu-Ala-Arg-p-nitroanilide
LDLa: low-density lipoprotein receptor class A
MSP: modular serine protease
PAP: PPO activating protease
PO: phenoloxidase
PPAE: PPO activating enzyme
PPAF: PPO activating factor
PPO: prophenoloxidase
PRRs: pattern recognition receptors
SAE: SPE activating protease
SPE: Spätzle processing enzyme.
