# Supplementary material for: Identification of a Conserved Prophenoloxidase Activation Pathway in Cotton Bollworm Helicoverpa armigera
Source: Front Immunol. 2020 May 5;11:785. doi: 10.3389/fimmu.2020.00785 (PMC7215089; doi:10.3389/fimmu.2020.00785)
Supplement: Supplementary file 1 [file Image_1.PDF]

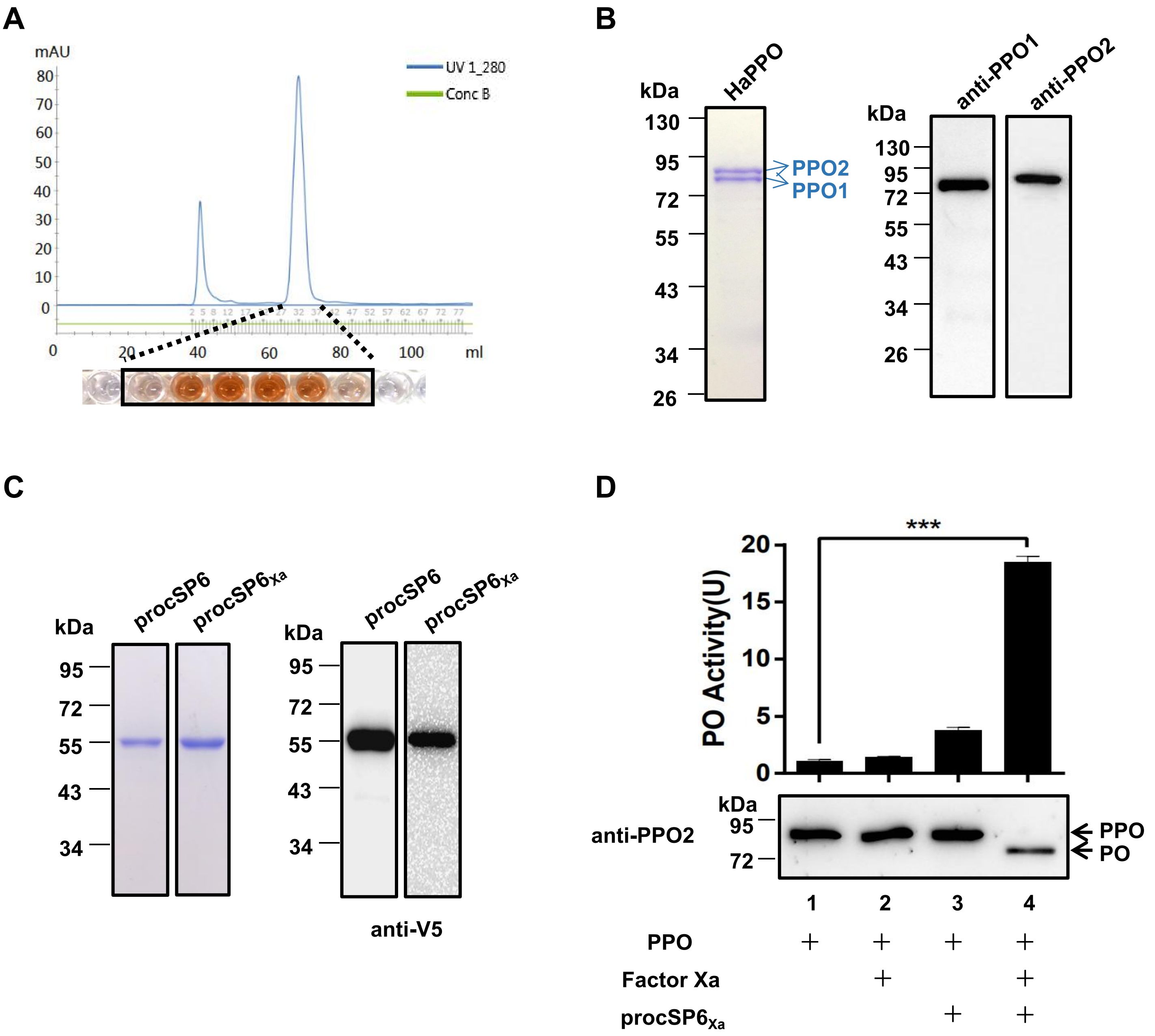

Figure S1. PPO purified from naïve 5th instar larvae is activated by cSP6$_{Xa}$. **(A)** Gel filtration chromatography of PPO. Two significant peaks were observed after gel filtration chromatography; fractions with CPC-activated PO activity are indicated. **(B)** Purified PPO detected by SDS-PAGE and immunoblotting analysis. **(C)** SDS-PAGE and immunoblot analysis of purified recombinant procSP6 and procSP6$_{Xa}$. Anti-V5 antibody was used to detect recombinant proteins by immunoblotting. **(D)** PPO is activated by cSP6. procSP6$_{Xa}$ (50 ng) was activated by factor Xa and then incubated with 100 ng or 300 ng PPO for 10 min. The mixtures containing 100 ng PPO (lower panel) were analyzed by immunoblotting using anti-PPO2 antibody (middle-panel) and the mixtures containing 300 ng PPO were used to measure PO activity (upper panel). PO activity was represented as mean ± SD of three independent experiments. Statistical analysis was performed using a t-tests analysis with GraphPad Prism. ***, $P < 0.001$.
