# Supplementary material for: Identification of a Conserved Prophenoloxidase Activation Pathway in Cotton Bollworm Helicoverpa armigera
Source: Front Immunol. 2020 May 5;11:785. doi: 10.3389/fimmu.2020.00785 (PMC7215089; doi:10.3389/fimmu.2020.00785)
Supplement: Supplementary file 2 [file Image_2.PDF]

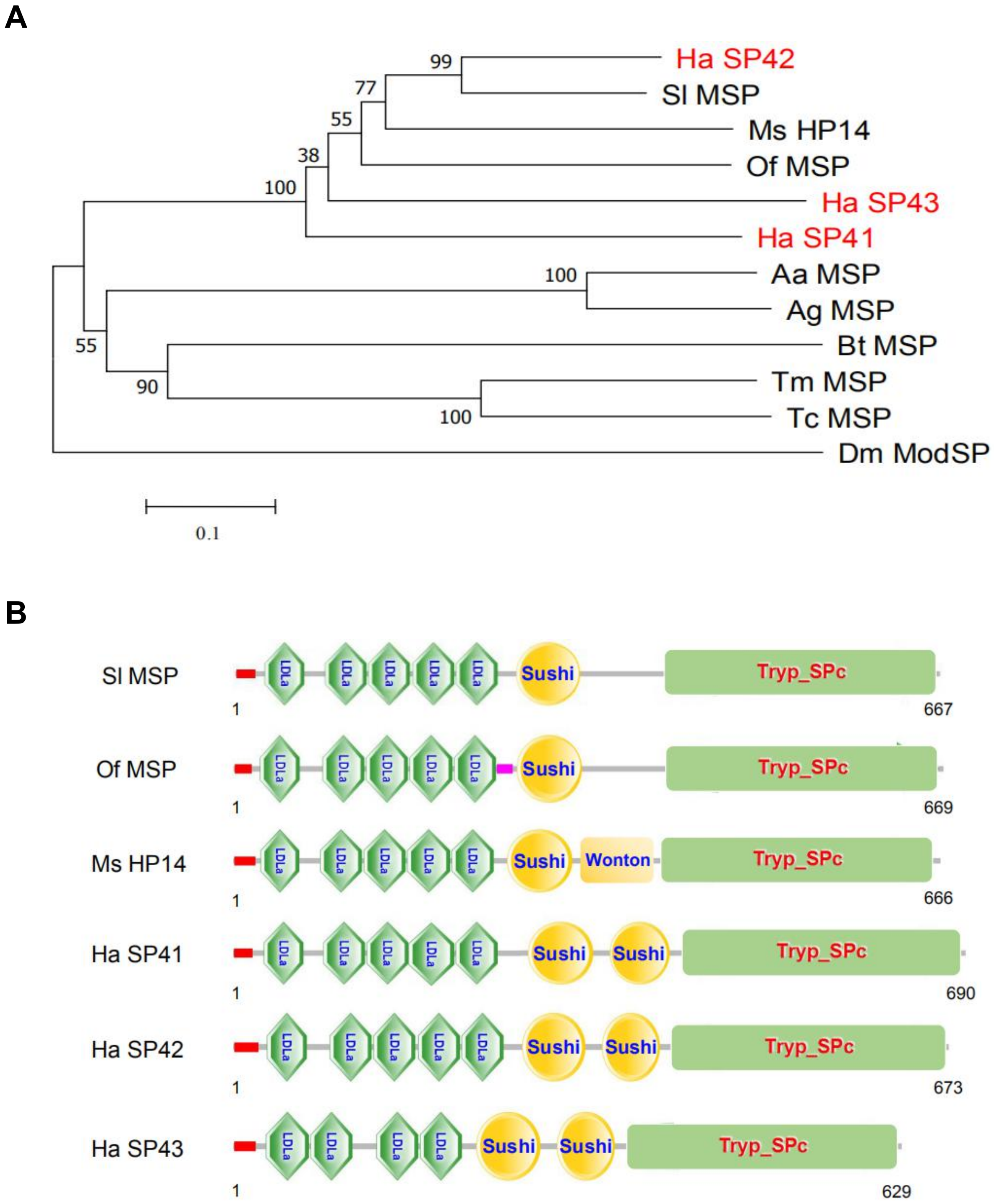

Figure S2. Analysis of modular SPs in *H. armigera*. **(A)** Phylogenitic analysis of modular SPs in *H. armigera*. The amino acid sequences of *Spodoptera litura* (Sl) MSP (XP_022819011.1), Ms HP14 (AAR29602.1), *Ostrinia furnacalis* (Of) MSP (XP_028166486.1), *Aedes aegypti* (Aa) MSP (XP_021707386.1), *Anopheles gambiae* (Ag) MSP (XP_321263.5), *Bombus terrestris* (Bt) MSP (XP_012175299.1), Tm MSP (BAG14264.1), *Tribolium castaneum* (Tc) MSP (XP_967486.1), Dm ModSP (NP_536776.2), and three Ha modular SPs were analyzed. Scale bar, 1.0 substitutions per site. **(B)** Domain architecture of Sl MSP, Of MSP, Ms HP14, Ha SP41, Ha SP42, and Ha SP43. Sl MSP and Of MSP both consisted of five LDLa, Sushi domain, and trypsin-like SP (Tryp_SPc). Ms HP14 consisted of five LDLa, Sushi domain,Wonton domain, and Tryp_SPc. For Ha SP41, five LDLa, two Sushi domain and one Tryp_SPc domain were indicated. All of the domains of Ha SP42 and Ha SP43 were also shown.
