# Supplementary material for: Identification of a Conserved Prophenoloxidase Activation Pathway in Cotton Bollworm Helicoverpa armigera
Source: Front Immunol. 2020 May 5;11:785. doi: 10.3389/fimmu.2020.00785 (PMC7215089; doi:10.3389/fimmu.2020.00785)
Supplement: Supplementary file 3 [file Image_3.PDF]

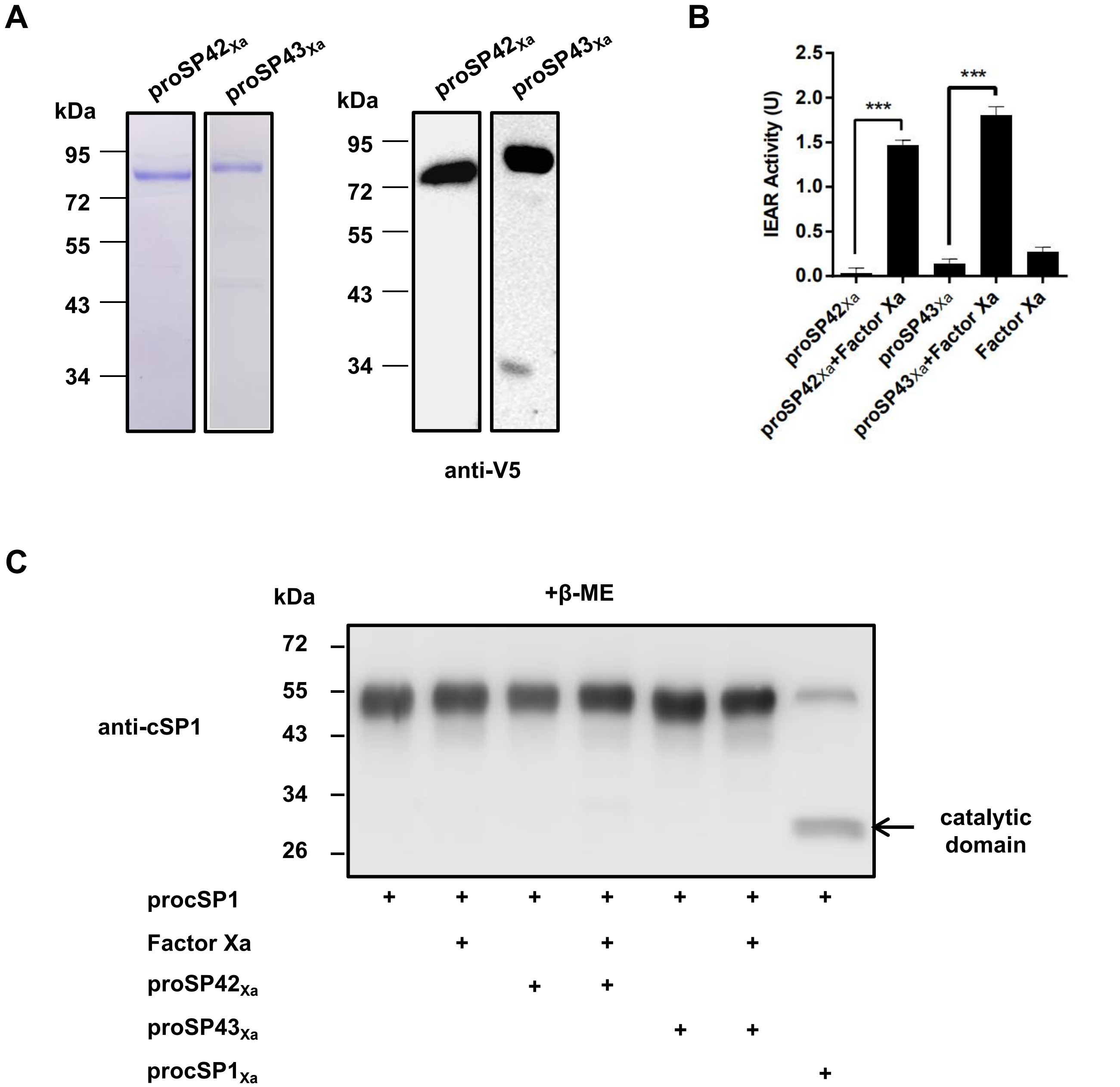

Figure S3. Testing the activation of procSP1 by SP42$_{Xa}$ and SP43$_{Xa}$. **(A)** SDS-PAGE and immunoblot analysis of purified recombinant proSP42$_{Xa}$ and proSP43$_{Xa}$. Anti-V5 antibody was used to detect recombinant proteins by immunoblotting. **(B)** Amidase activity of SP42$_{Xa}$ and SP43$_{Xa}$. Catalytic activity of activated SP42$_{Xa}$ (300 ng) and SP43$_{Xa}$ (300 ng) were detected using IEAR as a substrate. Statistical analysis was performed using a t-tests analysis with GraphPad Prism. ***, $P < 0.001$. **(C)** Factor Xa activated proSP42$_{Xa}$ or proSP43$_{Xa}$ (50 ng) was incubated with procSP1 (100 ng) for 1 h. The mixtures were subjected for SDS-PAGE under reducing condition (with β-ME) and analyzed by immunoblotting analysis using anti-cSP1 antibody.
