# Supplementary material for: Identification of a Conserved Prophenoloxidase Activation Pathway in Cotton Bollworm Helicoverpa armigera
Source: Front Immunol. 2020 May 5;11:785. doi: 10.3389/fimmu.2020.00785 (PMC7215089; doi:10.3389/fimmu.2020.00785)
Supplement: Supplementary file 4 [file Table_1.DOC]

| **Table S1.** Primers used for protein expression | | | |
| --- | --- | --- | --- |
| Function | Gene | Primers (5' to 3') | |
|  |  | Forward | Reverse |
| For protein expression in the *Drosophila* expression system | SP41 | CCGGAATTCTTCAGTGGTCAGGCAA | CCGCTCGAGTAAATATTGGTTTATA |
|  | cSP1 | CCGGAATTCTCAGTATGAAGGTGAAAC | CTAGTCTAGAATTAGGCCACACAATGC |
|  | cSP6 | CCGGAATTCCAAAATTCTTGCCAGACG | GAAGGAACAATAGTGGTCCTAATCC |
|  | cSPH11 | CCGGAATTCTCAGAAGAACAATGG | CTAGTCTAGAATACGTGTAAGTCTTAG |
|  | cSPH50 | CCGGAATTCTCAGTCGACCATTG | CTAGTCTAGAGTCCACATTTGATATCC |
|  | SP41_Xa_ | CCGGAATTCTTCAGTGGTCAGGCAAATTG  GTCGGAGGACTCCAGCCATAGAAGGCCGGGTGGCCGGTG | CAAGGCTGGCTGGCCACCGGCCACCCGGCCTTCTATGGCTGGAGTC  CCGCTCGAGTAAATATTGGTTTATAAAAATTCGATGTC |
|  | cSP1_Xa_ | CCGGAATTCTCAGTATGAAGGTGAAACGTGC  CAATAACTGTCATCATAAGATAGAAGGCCGGGTAGTCGCTGGTGAAG | CTTCACCAGCGACTACCCGGCCTTCTATCTTATGATGACAGTTATTG  CTAGTCTAGAATTAGGCCACACAATGCCCTCGATC |
|  | cSP6_Xa_ | CCGGAATTCTCAAAATTCTTGCCAGACGCCCAGC  CTGTGGTTTGGACAGCAGAATAGAAGGCCGGATCGTGGGAGGTACAGCG | CGCTGTACCTCCCACGATCCGGCCTTCTATTCTGCTGTCCAAACCAC  CTAGTCTAGAAGGAACAATAGTGGTCCTAATCCAGTC |
|  | cSPH11_Xa_ | CCGGAATTCTCAGAAGAACAATGGGGAC  GTTGGGCCAACCCAGATGGTATAGAAGGCCGGACAACCGGTGAAG | CATTTACTTCACCGGTTGTCCGGCCTTCTATACCATCTGGGTTGGC  CTAGTCTAGAATACGTGTAAGTCTTAGGGTCATAGC |
|  | cSPH50_Xa_ | CCGGAATTCTCAGTCGACCATTGATCCGAATATAC  GTCACTGGATGGGGCGTTATAGAAGGCCGGTTCGGAGAAGATGACAAC | GTTGTCATCTTCTCCGAACCGGCCTTCTATAACGCCCCATCCAGTGAC  CTAGTCTAGAGTCCACATTTGATATCCTGTAAGACGTC |
| For protein expression in the *E. coli* expression system | cSP6 | GCATGACTGGTGGACAGCAAAATTC | CTAGTTATTGCTCAGCGGTTAAGGAAC |
|  | cSPH11 | CCGGAATTCCAGAAGAACAATGG | CCGCTCGAGTATACGTGTAAGTCTTAG |
|  | cSPH50 | CCGGAATTCCAGTCGACCATTGATC | CCGCTCGAGTGTCCACATTTGATATC |
|  | PPO1 | CTAGTTATTGCTCAGCGGTCGGACG | CTAGTTATTGCTCAGCGGCCGCCTCTG |
|  | PPO2 | CTAGTTATTGCTCAGCGGGCCGACG | CTAGTTATTGCTCAGCGGTTAGTTGG |
